# Supplementary material for: Recurrence Pattern of Left Upper Lobectomies and Trisegmentectomies: Systematic Review and Meta-Analysis
Source: J Clin Med. 2025 Jun 19;14(12):4385. doi: 10.3390/jcm14124385 (PMC12194065; doi:10.3390/jcm14124385)
Supplement: Supplementary file 1 [file jcm-14-04385-s001.zip › jcm-3662253-supplementary.pdf]

Table S1: Sensitivity analysis for locoregional recurrence

Omitting study	OR	IC 95	P valor	tau2	tau	I^2
Omitting Iwasaki (2007)	0.8711	[0.6193; 1.2252]	0.43	< 0.0001	0.0015	0%
Omitting Soukasian (2012)	0.8713	[0.6215; 1.2215]	0.42	0	0	0%
Omitting Witte (2013)	0.8713	[0.6215; 1.2215]	0.42	0	0	0%
Omitting Aprile (2017)	0.7979	[0.5587; 1.1394]	0.21	0	0	0%
Omitting Zhou (2022)	0.8639	[0.6098; 1.2239]	0.41	0	0	0%
Omitting Nishikubo (2022)	0.8661	[0.6133; 1.2232]	0.41	0	0	0%
Omitting Tane (2024)	0.9304	[0.571; 1.516]	0.77	0.007	0.0834	0%
Omitting Aguinagalde (2024)	0.8552	[0.5888; 1.2423]	0.41	0	0	0%
Omitting McAllister (2024)	0.9477	[0.6666; 1.3474]	0.77	0	0	0%
Common effect model	0.8713	[0.6215; 1.2215]	0.42	0	0	0%

Table S2. Sensitivity analysis for distant recurrence

Omitting study	OR	IC 95	p valor	tau2	tau	I^2
Omitting Iwasaki (2007)	0.5775	[0.4055; 0.8224]	<0.01	0	0	0%
Omitting Soukasian (2012)	0.5805	[0.412; 0.818]	<0.01	0	0	0%
Omitting Witte (2013)	0.5805	[0.412; 0.818]	<0.01	0	0	0%
Omitting Aprile (2017)	0.6039	[0.4197; 0.8691]	<0.01	0	0	0%
Omitting Zhou (2022)	0.5299	[0.3645; 0.7705]	<0.01	0	0	0%
Omitting Nishikubo (2022)	0.5697	[0.3997; 0.812]	<0.01	0	0	0%
Omitting Tane (2024)	0.6393	[0.4006; 1.0203]	0.06	0	0	0%
Omitting Aguinagalde (2024)	0.5898	[0.407; 0.8547]	<0.01	0	0	0%
Omitting McAllister (2024)	0.5805	[0.412; 0.818]	<0.01	0	0	0%
Common effect model	0.5805	[0.4120; 0.8180]	<0.01	0	0	0%

Table S3. Sensitivity analysis for global recurrence

**Omitting study**	**OR**	**IC 95**	**P valor**	**tau2**	**tau**	**I^2**
Omitting Iwasaki (2007)	0.6542	[0.4987; 0.8583]	<0.01	0	0	0%
Omitting Soukasian (2012)	0.6548	[0.5022; 0.8538]	<0.01	0	0	0%
Omitting Witte (2013)	0.6548	[0.5022; 0.8538]	<0.01	0	0	0%
Omitting Aprile (2017)	0.6351	[0.4789; 0.8422]	<0.01	0	0	0%
Omitting Zhou (2022)	0.6391	[0.4833; 0.8449]	<0.01	0	0	0%
Omitting Nishikubo (2022)	0.6661	[0.5078; 0.8738]	<0.01	0	0	0%
Omitting Tane (2024)	0.619	[0.4228; 0.9063]	0.01	0	0	0%
Omitting Aguinagalde (2024)	0.6764	[0.5082; 0.9004]	<0.01	0	0	0%
Omitting McAllister (2024)	0.6781	[0.517; 0.8894]	<0.01	0	0	0%
Common effect model	0.6548	[0.5022; 0.8538]	<0.01	0	0	0%

Table S4. Sensitivity analysis for morbidity

Omitting study	OR	IC 95	P valor	tau2	tau	I^2
Omitting Iwasaki (2007)	0.9313	[0.7388; 1.174]	0.55	0.202	0.4494	64.72%
Omitting Soukasian (2012)	0.7843	[0.611; 1.0067]	0.06	0	0	0%
Omitting Witte (2013)	0.9661	[0.7653; 1.2197]	0.77	0.2229	0.4721	66.51%
Omitting Aprile (2017)	0.9591	[0.7543; 1.2197]	0.73	0.2409	0.4909	66.84%
Omitting Zhou (2022)	1.0009	[0.7824; 1.2805]	0.99	0.2288	0.4783	65.13%
Omitting Nishikubo (2022)	0.9802	[0.7783; 1.2346]	0.86	0.1934	0.4397	64.09%
Omitting Tane (2024)	1.0546	[0.8148; 1.3649]	0.69	0.216	0.4648	61.94%
Omitting Aguinagalde (2024)	0.9584	[0.7517; 1.2218]	0.73	0.2437	0.4937	66.85%
Omitting McAllister (2024)	0.9857	[0.7735; 1.2561]	0.91	0.232	0.4816	65.86%
Common effect model	0.9548	[0.7607; 1.1986]	0.69	0.1959	0.4426	62.1%

Table S5. Sensitivity analysis for the length of stay

**Omitting study**	**OR**	**IC 95**	**P valor**	**tau2**	**tau**	**I^2**
Omitting Iwasaki (2007)	-0.94	[-1.2568;-0.6294]	0	0.1283	0.3581	29.22%
Omitting Soukasian (2012)	-0.89	[-1.2137;-0.5642]	0	0.0872	0.2952	27.29%
Omitting Witte (2013)	-0.94	[-1.2519;-0.6214]	0	0.1313	0.3623	39.83%
Omitting Aprile (2017)	-0.91	[-1.2372;-0.5803]	0	0.1956	0.4423	37.48%
Omitting Zhou (2022)	-1.27	[-1.7109;-0.8329]	0	0.0213	0.1458	0%
Omitting Nishikubo (2022)	-0.90	[-1.2138;-0.5793]	0	0.057	0.2387	0%
Omitting Tane (2024)	-0.94	[-1.2568;-0.6294]	0	0.1283	0.3581	29.22%
Omitting Aguinagalde (2024)	-0.93	[-1.2434;-0.6076]	0	0.1323	0.3637	37.7%
Omitting McAllister (2024)	-0.92	[-1.2934;-0.5445]	0	0.2883	0.5369	40.64%
Common effect model	-0.9431	[-1.2568; -0.6294]	< 0.0001	0.1283	0.3581	29.2%
